# Editorial: Use of computerized gait analysis in neurological pathologies

**DOI:** 10.3389/fnhum.2022.1078539

**Published:** 2022-12-07

**Authors:** Simone Carozzo, Carmelo Chisari, Marco Iosa

**Affiliations:** ^1^Research in Advanced Neurorehabilitation (RAN), Sant'Anna Crotone Institute, Crotone, Italy; ^2^Department of Translational Research and New Technology in Medicine and Surgery, University of Pisa, Pisa, Italy; ^3^Department of Psychology, Faculty of Medicine and Psychology, Sapienza University of Rome, Rome, Italy; ^4^IRCCS Santa Lucia Foundation, Rome, Italy

**Keywords:** gait analysis, neurological pathologies, computerized gait analysis, machine learning, rehab

The interest in computer-assisted quantitative gait evaluation sharply increased sharply in recent years (Figure 1). Its widespread use provides evidence of applicability to obtain objective and comparable measures supplementing clinical scales and subjective judgments.

**Figure 1:**
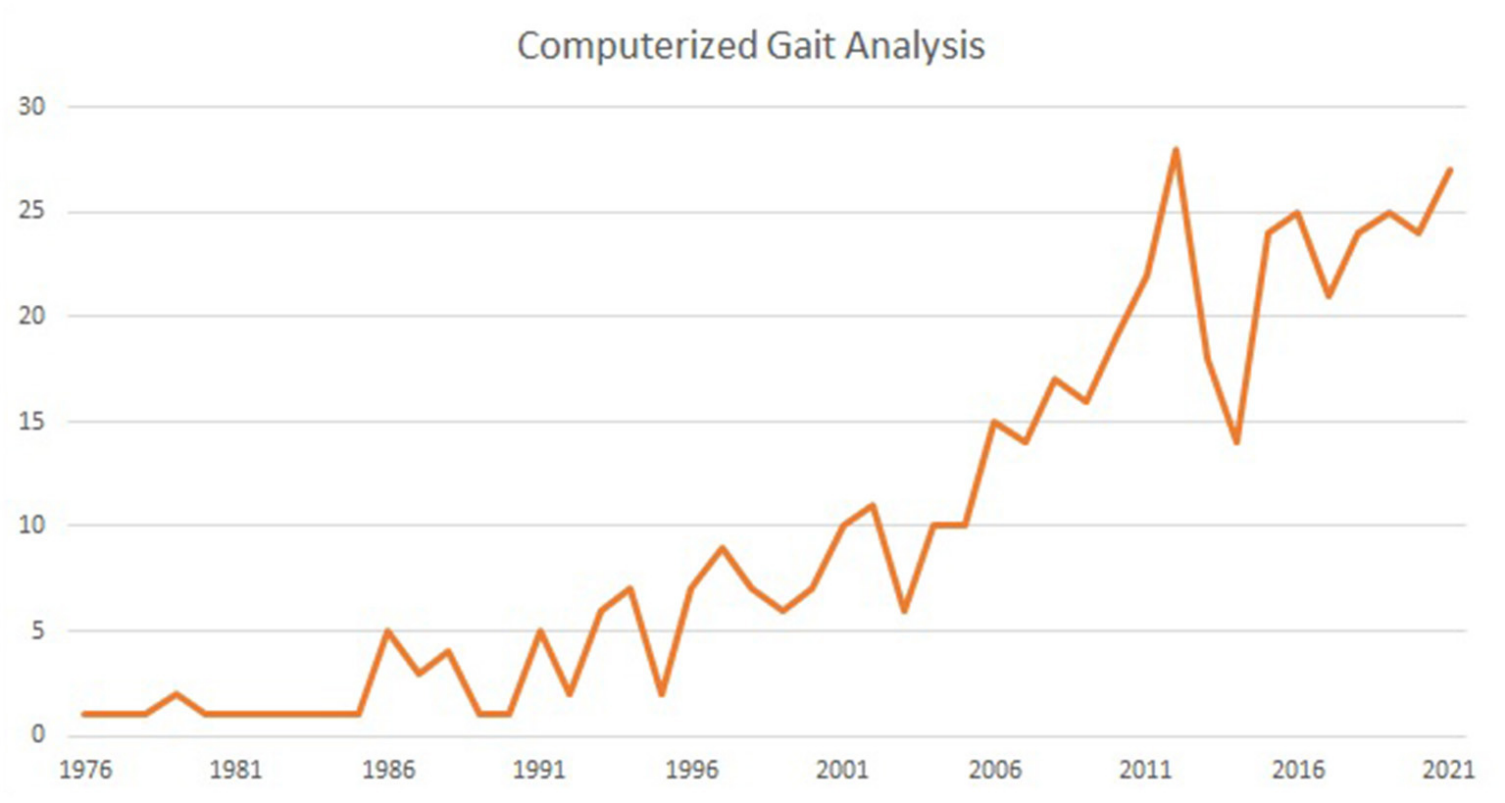
The increment of publications related to “computerized gait analysis” as reported on Pubmed at November 2022.

The aim of this Research Topic was to collect papers exploring the utility of computerized gait analysis for exploring the impact of neurological diseases on gait functions. We have selected 13 articles that have been found to be of high quality for publication in this Research Topic. These papers covered different aspects related to protocols and technologies, and many different pathologies (stroke, cerebral palsy, Parkinson's disease, multiple sclerosis, spinal cord injury, Charcot Marie Tooth disease, Duchenne muscular dystrophy).

Systematic research has implemented procedures and paradigms generating a large number of potential variables for kinematic analysis; some debate on their relevance and proper selection is going on; several algorithms and equipment configurations to automatically identify the timing of gait events are being tested to comply with the clinician's needs. Whether individual markers of combinations of markers and their position on different body districts can influence the accuracy of the algorithms and therefore, the clinical and scientific findings remain unclear (Visscher et al.).

Usual gait analysis often reports the kinematics of hip, knee and ankle, but the influence of the pelvis as a biomechanical constraint during gait also needs to be deeply investigated (Favetta et al.).

Multiple Sclerosis has been systematically studied by means of kinematic analysis, partly due to its being characterized by the fatigue progressing with the disease worsening. This fatigue in walking can be studied through an antiphase pattern of limbs coordination while seated when movements of lower are performed leg as quickly as possible (Goetschalckx et al.). Over the years, thanks to the increasing collaboration between clinicians and complex systems analysts, individual “indices' have been identified to characterize the evolution of various diseases and to quantify and monitor the patients; motor recovery, in order to verify the effectiveness of medical and physiotherapic treatments.

In the study of chronic post-stroke hemiparetic patients, for instance, the range of motion of the ankle, spasticity of the triceps sural, walking speed, the scales related to assistance and the limitation in the subjects; social participation have been taken in to consideration (Mazzoli et al.). Or again for post-stroke subjects a set of kinematic variables that are sufficient to evaluate gait, thus being able to substantially facilitate future diagnosis and monitoring of rehabilitation progress (Nedergård et al.).

One of the main developers and computerized systems analysts; challenge in the analysis of gait kinematics is to create indicators as reliable as the clinical scales in use. The main options are basically two:

1- to create analysis procedures and verify their compatibility with existing scales; or 2- to identify indicators able to replace the same scales with comparable or better reliability. An example in this regard is the attempt to provide objective interpretations of the observational gait assessment based on the Wisconsin Gait Scale (WGS) used to assess walking quality in post-stroke hemiplegic subjects. Computer-assisted investigations proved able to provide useful, low-cost and timely feedback to monitor treatments and the evolution of the disease (Guzik et al.).

For a long time, especially in Europe, the gait of subjects with infantile cerebral palsy have been examined by assessing the clinical relevance of fluctuations in the regularity of gait patterns between repetitive cycles. The approach is congruent with sensorimotor system organization, with gait fluctuation and regularity influenced by factors such as age and pathology, and supports the interpretation of clinical gait analysis and therapeutic decision making (Tabard-Fougère et al.).

3D gait analysis allows compare systematically and without human error the gait patterns of different neuromotor conditions: normal gait, cerebral palsy (CP), Charcot Marie's tooth (CMT) and Duchenne muscular dystrophy (DMD). These comparisons can be of use in the correct interpretation of gait clinical pattern (Minosse et al.).

The Gait Deviation Index (GDI), a multivariate measure of the overall gait pathology based on 15 gait characteristics derived from three-dimensional kinematic data, proved an indicator most suitable of application with good efficacy in the evaluation of cerebral (CP), post-stroke hemiparetic gait, Duchenne muscular dystrophy and Parkinson's disease, and its clinical significance also in other pathologies such as spinal cord injury (SCI) is being tested (Sinovas-Alonso et al.). But also, an identification of a core set of gait features could be helpful for clinicians to support treatment decision-making processes related to children with CP (van der Krogt et al.).

The use of machine learning algorithms is increasingly frequent in instrumental diagnostics and evaluation, even in the field of computerized motion analysis. Its purpose is to treat variables and indices extrapolated from the various tools; it allows to classify and evaluate patients in remote and help doctors in making decisions and monitor treatment and follow-up, without depending on the administration of scales or on personal clinical experience. For example, to classify patients with Parkinson's disease in a useful and accurate way, they use low-cost portable devices (Muñoz-Ospina et al.). Also, children with cerebral palsy could benefit from a machine learning approach for identifying the intertwine between motor control and gait kinematics after orthopedic surgery (Steele and Schwartz).

Three-dimensional gait analysis can provide parameters to study the effectiveness of clinical trials aimed at preserving walking, as data can be recorded covering the entire walking period, by performing a so-called mixed cross-sectional longitudinal study, without possible variations in evaluations due to the change of the test administrator or clinical evaluator. As in treatment in children with Duchenne muscular dystrophy (DMD) (Vandekerckhove et al.).

## Author contributions

SC chose the other two guest editors and the specialists to send the proposal to participate in the Research Topic, carried out the task of accepting some works after the reviews of the reviewers, and wrote editorial. CC and MI accepted some jobs after the reviews and editorial revisions. All authors contributed to the article and approved the submitted version.

